# Establishing a Risk Prediction Model for Atherosclerosis in Systemic Lupus Erythematosus

**DOI:** 10.3389/fimmu.2021.622216

**Published:** 2021-04-16

**Authors:** Haiping Xing, Haiyu Pang, Tian Du, Xufei Yang, Jing Zhang, Mengtao Li, Shuyang Zhang

**Affiliations:** ^1^State Key Laboratory of Complex Severe and Rare Diseases, Department of Cardiology, Peking Union Medical College Hospital, Peking Union Medical College and Chinese Academy of Medical Sciences, Beijing, China; ^2^State Key Laboratory of Complex Severe and Rare Diseases, Medical Research Center, Peking Union Medical College Hospital, Peking Union Medical College and Chinese Academy of Medical Sciences, Beijing, China; ^3^School of Medicine, Tsinghua University, Beijing, China; ^4^Department of Breast Oncology, Sun Yat-sen University Cancer Center, Guangzhou, China; ^5^Department of Diagnostic Ultrasound, Peking Union Medical College Hospital, Peking Union Medical College and Chinese Academy of Medical Sciences, Beijing, China; ^6^Key Laboratory of Rheumatology and Clinical Immunology, Department of Rheumatology, National Clinical Research Center for Dermatologic and Immunologic Diseases, Ministry of Science & Technology, Ministry of Education, Peking Union Medical College Hospital, Peking Union Medical College and Chinese Academy of Medical Sciences, Beijing, China

**Keywords:** systemic lupus erythematosus, atherosclerosis, prediction model, RNA-Seq, differential gene analysis

## Abstract

**Background and aims:** Patients with systemic lupus erythematosus (SLE) have a significantly higher incidence of atherosclerosis than the general population. Studies on atherosclerosis prediction models specific for SLE patients are very limited. This study aimed to build a risk prediction model for atherosclerosis in SLE.

**Methods:** RNA sequencing was performed on 67 SLE patients. Subsequently, differential expression analysis was carried out on 19 pairs of age-matched SLE patients with (AT group) or without (Non-AT group) atherosclerosis using peripheral venous blood. We used logistic least absolute shrinkage and selection operator regression to select variables among differentially expressed (DE) genes and clinical features and utilized backward stepwise logistic regression to build an atherosclerosis risk prediction model with all 67 patients. The performance of the prediction model was evaluated by area under the curve (AUC), calibration curve, and decision curve analyses.

**Results:** The 67 patients had a median age of 42.7 (Q1–Q3: 36.6–51.2) years, and 20 (29.9%) had atherosclerosis. A total of 106 DE genes were identified between the age-matched AT and Non-AT groups. Pathway analyses revealed that the AT group had upregulated atherosclerosis signaling, oxidative phosphorylation, and interleukin (IL)-17-related pathways but downregulated T cell and B cell receptor signaling. Keratin 10, age, and hyperlipidemia were selected as variables for the risk prediction model. The AUC and Hosmer–Lemeshow test *p*-value of the model were 0.922 and 0.666, respectively, suggesting a relatively high discrimination and calibration performance. The prediction model had a higher net benefit in the decision curve analysis than that when predicting with age or hyperlipidemia only.

**Conclusions:** We built an atherosclerotic risk prediction model with one gene and two clinical factors. This model may greatly assist clinicians to identify SLE patients with atherosclerosis, especially asymptomatic atherosclerosis.

## Background

Systemic lupus erythematosus (SLE) is a chronic autoimmune disease that affects multiple systems including the cardiovascular system (1). The incidence of cardiovascular events in SLE patients is significantly higher than that in the general population (2–4). Among females aged 35–44 years, SLE patients are 50 times more likely to develop myocardial infarction than healthy control subjects (4). Cardiovascular disease (CVD) is one of the most common causes of death in SLE patients, accounting for 25–30% of all deaths (5–7).

Atherosclerosis is the main cause of CVD. Patients with SLE also exhibit a higher prevalence of atherosclerosis than the general population (8, 9). In SLE, atherosclerosis is related to both traditional risk factors, such as age, sex, hyperlipidemia, and SLE-related features, such as disease duration, disease activity, and medications (3, 10). At the gene expression level, studies have determined that multiple proteins, including inflammatory cytokines [interleukin (IL)-1, IL-6, IL-10, interferon (IFN)-γ, tumor necrosis factor (TNF)-α, transforming growth factor (TGF)-β] and vascular endothelial related molecules [high-mobility group box protein (HMGB)1, vascular cell adhesion molecule (VCAM)-1, intercellular adhesion molecule (ICAM)-1, vascular endothelial growth factor (VEGF)], are closely related to atherosclerosis in SLE patients (10, 11). High-throughput sequencing is currently being applied to investigate atherosclerosis in SLE (12–14). Although some interesting findings have been reported, the number of studies is relatively small, and the biological pathways that contribute to the increased atherosclerosis in SLE are not fully understood.

Early identification of SLE patients with atherosclerosis, which is often asymptomatic at the early stage, is important for prevention of atherosclerosis progression and future CVD. However, traditional prediction models, such as the Framingham risk score, largely underestimate the prevalence of atherosclerosis or cardiovascular events in SLE patients (15, 16), and studies on specific prediction models for atherosclerosis in SLE patients are limited. To the best of our knowledge, the only reported prediction model is the Predictors of Risk for Elevated Flares, Damage Progression, and Increased Cardiovascular Disease in Patients with SLE (PREDICTS) model developed by McMahon et al. (17). Composed of four biomarkers (leptin, soluble TWEAK, homocysteine, and proinflammatory high-density lipoprotein), age, and diabetes, the PREDICTS model has relatively high sensitivity (89%) and specificity (79%). Nevertheless, the four biomarkers were derived from a limited number of candidate biomarkers. Thus, it remains unclear whether combinations of untested biomarkers or genes can achieve better prediction accuracy. The objective of this study was to use RNA sequencing (RNA-seq) analysis to investigate the underlying mechanisms of atherosclerosis in SLE patients and to establish a diagnositic prediction model for atherosclerosis in SLE that combines high-throughput sequencing data and clinical risk factors.

## Methods

### Study Population and Age Matching

This cross-sectional study enrolled patients from the Chinese SLE Treatment and Research Group (CSTAR) registry (18) who presented to the outpatient clinic of the Peking Union Medical College Hospital between June 2019 and September 2019. All patients fulfilled the following criteria: (a) age >18 years and (b) meeting the 2012 Systemic Lupus erythematosus International Collaborating Clinics (SLICC) classification criteria (19). The exclusion criteria were as follows: (a) active infection, (b) presence of a tumor or other connective tissue disease, (c) currently in a period of moderate to severe SLE [SLE Disease Activity Index 2000 (SLEDAI-2K) ≥10], and (d) no history of CVD before the diagnosis of SLE. Subsequently, 1:1 age matching (±6 years) was performed for SLE patients with atherosclerosis (AT group) and without (Non-AT group) atherosclerosis. A 66-year-old patient was unable to be matched because the SLE AT group was significantly older than the SLE Non-AT group. There were five patients from the AT group who had several age- and sex-matching patients from the non-AT group. In such condition, we also took body mass index (BMI) and SLEDAI into consideration and excluded the other patients.

### Clinical and Laboratory Data

Venous blood was extracted between 8:00 and 9:00 A.M. after an 8-h fasting. All laboratory tests were conducted at the Department of Clinical Laboratory, Peking Union Medical College Hospital. Complete blood counts were analyzed using an automated hematology system (Model XE2100; Sysmex Co., Kobe, Japan). Blood biochemistry data were measured by an automatic biochemistry analyzer (AU5821; Beckman Coulter, Miami, FL, US), including fasting blood glucose, creatinine (Cr), uric acid (UA), total cholesterol (TC), triglyceride (TG), high-density lipoprotein cholesterol (HDL-C), low-density lipoprotein cholesterol (LDL-C), complement 3, complement 4, and hypersensitive C-reactive protein (hs-CRP). Glycated hemoglobin A1c (HbA1c) was examined with a fully automated glycated hemoglobin analyzer (Variant II; Bio-Rad, Hercules, CA, US). Cardiac enzymes including creatine kinase, creatine kinase isoenzyme, cardiac troponin I, and N-terminal pro-B-type natriuretic peptide were evaluated by a fully automated integrated biochemical analyzer (Dimension EXL; Siemens, Erlangen, Germany). Anti-dsDNA antibodies (IgG type) were measured by indirect immunofluorescence and ELISA (EA1571-9601G; EUROIMMUN, Lubeck, Germany). Indirect immunofluorescence >1:5 or ELISA >100 IU/ml was defined as positive. The erythrocyte sedimentation rate (ESR) was also measured.

Clinical data were collected from the patients by physicians. These data included the following: (a) traditional CVD risk factors: age, sex, history of hypertension, hyperlipidemia, and diabetes, BMI, history of smoking, family history of early-onset CVD (men aged <55 years and women aged <65 years in first-degree relatives), menopause history; (b) history of CVD, current use of aspirin, statin, and angiotensin-converting enzyme inhibitor/angiotensin receptor blocker; (c) SLE-related CVD risk factors: disease course, cumulative dose of prednisone in the previous 1 year, current use of immunosuppressants, and the SLICC/ACR Damage Index and SLEDAI-2K score (20, 21).

The brachial-ankle pulse wave velocity (baPWV) was quantified using a non-invasive vascular screening device (VP-2000, Omron-Colin, Tokyo, Japan) as described by Ming Ding et al. (22). The carotid intima media thickness (CIMT) was measured by a doctor specialized in diagnostic ultrasound. The detection range included the region 1 cm distal to the common carotid artery, 1 cm to the carotid bifurcation, and 1 cm to the proximal end of the internal carotid artery (14). For each patient, the maximum CIMT values for three sites on the left and right sides were recorded. Atherosclerosis was defined as CIMT ≥1.2 mm or a history of CVD after diagnosis of SLE (23).

### RNA Sequencing and Differential Gene Analysis

RNA was extracted from peripheral venous blood using a PAXgene Blood RNA purification kit (BD Bioscience, Franklin Lakes, NJ, USA). The integrity and concentration of the RNA were determined by an RNA Nano 6000 Kit (Agilent Technologies, Santa Clara, CA, USA). Paired-end RNA-seq was carried out on an Illumina HiSeq 2500 platform at Annoroad Gene Technology Co., Ltd. (Beijing, China). Salmon software (v1.14.1) was used to align the raw RNA-seq data to the reference genome hg38 (Ensemble version 84) to obtain gene counts (24). To remove the potential effect of age on the selection of differentially expressed (DE) genes, differential expression analysis was performed using R package DESeq2 (v.1.22.2) with age-matched AT and Non-AT groups. Absolute fold change >1.5 and *p* < 0.01 were used as cutoffs to select DE genes.

Heatmaps of the DE genes were constructed in R using the Heatmap.3 package. To generate the heatmap, raw gene counts from the 67 samples were normalized with a variance-stabilizing transformation (vst) using the DESeq2 package. The Euclidean distances between samples and genes were calculated and visualized by complete-linkage clustering.

### Pathway Analysis

Pathway analysis was conducted with Ingenuity Pathways Analysis (IPA v01-12; Qiagen, Hilden, Germany) using the upregulated and downregulated DE genes. Pathways with *p* < 0.05 were defined as upregulated or downregulated pathways.

Gene set enrichment analysis (GSEA v.4.0.3; Broad Institute, USA) was performed on selected pathways using the *GseaPreranked* function. All genes were ranked by their log_2_(fold change). “Enrichment Statistic” and “Min Size: exclude smaller sets” were set as “classic” and “5,” respectively. All other settings were default. Pathways with *p* < 0.05 and Enrichment Score > 0 or < 0 were considered as upregulated and downregulated pathways, respectively. The 186 Kyoto Encyclopedia of Genes and Genomes (KEGG) pathways were downloaded from the Molecular Signatures database (MSigDB, v7.0, Broad Institute). The immune cell signatures reported by Davoli et al. (25) were used to assess the enrichment of different immune cell lineages. Atherosclerosis signatures were obtained from previous studies by Huang et al. (26) and Nuhrenberg et al. (27).

### Statistical Analysis

All statistical analyses were conducted with the R software (v.3.6.2). For the AT and Non-AT groups, data were presented as median and quartiles (Q1–Q3) for continuous variables and as a percentage (n/N) for categorical variables. Comparisons between the two groups were conducted by the Wilcoxon rank-sum test and chi-square test for continuous variables and categorical variables, respectively.

**Figure 1:**
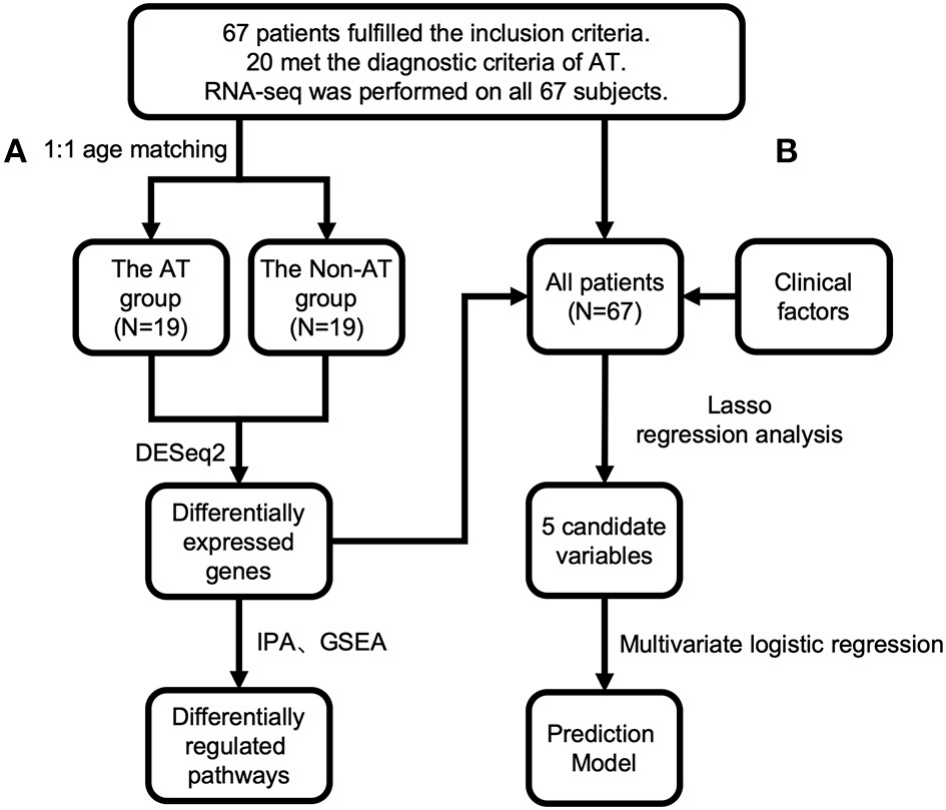
Study design and procedures of prediction model building. Sixty-seven patients fulfilled the inclusion criteria, among whom 20 had atherosclerosis (AT) by our definition. RNA sequencing (RNA-seq) was performed on all 67 patients. **(A)** For the differential expression analysis, 1:1 age-matched systemic lupus erythematosus (SLE) AT group (*n* = 19) and SLE Non-AT group (*n* = 19) were used. Ingenuity Pathway Analysis (IPA) and gene set enrichment analysis (GSEA) were performed with differentially expressed (DE) genes. **(B)** All 67 samples were used to build the prediction model for atherosclerosis. DE genes from **(A)** and clinical atherosclerosis risk factors were used as candidate variables for LASSO selection. Five variables were selected. A multivariate logistic regression was further applied to the five variables. Variables were excluded sequentially by the highest *p*-value in the multivariate logistic regression until all remaining variables had significant *p*-values. Three final variables were selected to build the final prediction model with multivariate logistic regression.

The methods described in this article are in accordance with the Transparent Reporting of a multivariable prediction model for Individual Prognosis Or Diagnosis (TRIPOD) statement (28). A prediction model was developed with all 67 SLE patients (*n* = 67). The logistic least absolute shrinkage and selection operator (LASSO) regression was implemented with R-package glmnet (v3.0-2). DE genes (vst normalized expression by DESeq2) and clinical risk factors for atherosclerosis (marked with # in Table 1) were used as candidate variables for LASSO selection (Figure 1). A 6-fold cross validation was used in the LASSO regression. Five candidate variables were selected by LASSO regression and further entered the multivariate logistic regression performed with R package “stat” (v3.6.2). Variables were sequentially excluded by the highest *p*-value in the multivariate logistic model until all the remaining variables had significant *p*-values. All *p*-values were two-sided, with the significance level set at 0.05. Receiver operating characteristic (ROC) curve and calibration curve were plotted using the “pROC” (v1.16.1) and “rms” (v5.1-4) packages, respectively. The 95% confidence interval of the area under the curve (AUC) was calculated by bootstrap resampling (500 times) of all SLE patients. The calibration curve was plotted with 1,000 times bootstrap resampling using “calibrate” function in the R package “rms.” The calibration performance of the model was tested by the Hosmer–Lemeshow goodness-of-fit test using R package “ResourceSelection” (v0.3-5). A decision curve was generated by the “rmda” package (v1.6) with “population.prevalence” set as 0.37, reflecting the prevalence of atherosclerosis in SLE patients reported by Roman et al. (8).

**Table 1:** Demographics and clinical features of age-matched SLE atheroslerosis and non-atherosclerosis groups.

**Factors**	**AT**	**Non-AT**	***p*-value**
	**(*N* = 19)**	**(*N* = 19)**	
Age (years)	52.2 (48.5–57.2)	48.4 (45.9–51.8)	0.199^#^
Male, n (%)	1 (5.3)	1 (5.3)	1.000^#^
BMI (kg/m^2^)	24.5 (23.6–26.7)	23.1 (21.9–24.5)	0.122^#^
Disease duration of SLE, (years)	10.1 (7.9–17.9)	13.6 (9.1–16.5)	0.661^#^
Ever smoker, n (%)	3 (15.8)	1 (5.3)	1.000^#^
Menopausal status, n (%)	14 (77.8)	15 (83.3)	1.000
Hypertension, n (%)	8 (42.1)	4 (21.1)	0.295^#^
Hyperlipidemia, n (%)	11 (57.9)	2 (10.5)	0.006^*^^#^
Diabetes mellitus, n (%)	2 (10.5)	2 (10.5)	1.000^#^
Family history of early onset CVD, n (%)	9 (47.4)	3 (15.8)	0.081^#^
Coronary heart disease, n (%)	3 (15.8)	0 (0)	0.229^#^
Stroke, n (%)	1 (5.3)	0 (0)	1.000^#^
TC (mmol/L)	4.23 (4.02–4.79)	3.99 (3.56–4.45)	0.287
TG (mmol/L)	1.15 (0.84–1.31)	1.07 (0.94–1.28)	0.884
HDL-C (mmol/L)	1.21 (1.11–1.59)	1.19 (1.12–1.36)	0.861
LDL-C (mmol/L)	2.46 (1.91–2.98)	2.25 (1.89–2.76)	0.414
WBC (×10^9^)	5.18 (3.92–7.50)	4.75 (4.45–5.80)	0.595
NEUT (×10^9^)	3.18 (1.86–4.50)	3.22 (2.40–3.79)	0.879
LYM (×10^9^)	1.82 (1.14–2.16)	1.31 (1.23–1.9)	0.447
PLT (×10^9^)	215 (148–254)	173 (157–237)	0.750
HbA1c (%)	5.4 (5.3–5.9)	5.5 (5.4–5.6)	0.626
FBG (mmol/L)	4.7 (4.6–5.0)	4.6 (4.5–4.9)	0.177
Cr (μmol/L)	64 (61–73)	59 (55–64)	0.064
UA (μmol/L)	280 (243–331)	288 (264–321)	0.563
C3 (g/L)	0.972 (0.925–1.208)	0.981 (0.846–1.126)	0.603
C4 (g/L)	0.181 (0.144–0.200)	0.146 (0.121–0.183)	0.255
ESR (mm/h)	11 (9–22)	19 (10–31)	0.367
hs-CRP (mg/L)	1.04 (0.67–3.81)	1.66 (0.74–2.23)	0.907
CK (U/L)	71 (57–92)	84 (64–95)	0.521
CK-MB (μg/L)	0.5 (0.5–0.6)	0.6 (0.5–0.7)	0.488
cTnI (μg/L)	<0.017	<0.017	0.343
NT-proBNP (pg/ml)	55 (37–95)	48 (35–72)	0.640
Anti-dsDNA antibodies, n (%)	7 (36.8)	9 (47.4)	0.742
Left CIMT (mm)	1.59 (0.69–1.84)	0.75 (0.64–0.82)	0.013^*^
Right CIMT (mm)	1.16 (0.77–1.76)	0.68 (0.58–0.80)	<0.001^*^
Left baPWV (cm/s)	1,509 (1,294–1,724)	1,347 (1,244–1,511)	0.168
Right baPWV (cm/s)	1,519 (1,300–1,704)	1,329 (1,220–1,498)	0.170
SLEDAI	2 (0–2)	2 (0–2)	0.827^#^
SLICC/ADI	0 (0–1)	0 (0–0)	0.076^#^
Aspirin, n (%)	5 (26.3)	2 (10.5)	0.403^#^
Statins, n (%)	6 (31.6)	0 (0)	0.026^*^^#^
ARB/ACEI, n (%)	6 (31.6)	3 (15.8)	0.445^#^
Corticosteroids, n (%)	11 (57.9)	9 (47.4)	0.745^#^
Current use of prednisone (mg)	2.5 (0–5.0)	0 (0–3.1)	0.456^#^
12-month cumulative prednisone (g)	0.91 (0–1.83)	0 (0–1.37)	0.567^#^
Hydroxychloroquine, n (%)	17 (89.5)	16 (84.2)	1.000^#^
Cyclophosphamide, n (%)	2 (10.5)	0 (0)	0.468^#^
Azathioprine, n (%)	2 (10.5)	0 (0)	0.468^#^
Cyclosporine, n (%)	0 (0)	0 (0)	1.000^#^
*Tripterygium wilfordii*, n (%)	0 (0)	1 (5.3)	1.000^#^
Mycophenolate mofetil, n (%)	0 (0)	4 (21.1)	0.113^#^

*AT, atherosclerosis; Non-AT, patients without atherosclerosis; TC, total cholesterol; TG, triglyceride; LDL-C, low-density lipoprotein cholesterol; HDL-C, high-density lipoprotein cholesterol; WBC, white blood cell; NEUT, neutrophil; LYM, lymphocyte; PLT, platelet; HbA1c, glycated hemoglobin A1c; FBG, fasting blood glucose; Cr, creatinine; UA, uric acid; C3, complement 3; C4, complement 4; ESR, erythrocyte sedimentation rate; hs-CRP, high-sensitivity C-reactive protein; CK, creatine kinase; CK-MB, creatine kinase isoenzyme; cTnI, cardiac troponin I; NT-proBNP, N-terminal pro-B-type natriuretic peptide; dsDNA, double-strand DNA; CIMT, carotid intima media thickness; baPWV, the brachial-ankle pulse wave velocity; SLEDAI, systemic lupus erythematosus disease activity index 2000; SLICC/ADI, Systemic Lupus International Collaborating Clinics/ACR Damage Index; ARB/ACEI, angiotensin-converting enzyme inhibitor/angiotensin receptor blocker. Data were presented as median and quartiles (Q1–Q3) for continuous variables and as percentages (n/N) for categorical variables. Comparisons between the two groups were conducted with Wilcoxon rank-sum test and chi-square test for continuous variables and categorical variables, respectively*.

* *p < 0.05. Clinical factors used for LASSO selection was labeled with #*.

## Results

### Comparisons Between the Age-Matched AT and Non-AT Groups

A total of 67 patients fulfilled the inclusion criteria and were enrolled in the study. The median (Q1–Q3) age of all subjects was 42.7 (range 36.6–51.2) years with a median disease duration of 10.0 (6.6–15.2) years (Supplementary Table 1). Sixty-three (94%) patients were female, and 20 (29.9%) fulfilled the definition of atherosclerosis in our study. The AT group was significantly older than the Non-AT group (Supplementary Table 2).

Among the 67 patients, 19 pairs of patients from the AT and Non-AT groups were matched by age to remove the potential effect of age in screening DE genes. The median ages in the age-matched AT and Non-AT groups were 52.2 (48.5–57.2) years and 48.4 (45.9–51.8) years (*p* = 0.199, Wilcoxon rank-sum test; Table 1), respectively. Compared with the age-matched Non-AT group, the prevalence of hyperlipidemia in the AT group was significantly increased (*p* = 0.006), and the percentage of statin use was also higher (*p* = 0.026), while no significant differences were observed in TC, TG, HDL-C, and LDL-C. The inconsistency between hyperlipdimia and blood lipids could be attributed to the statin use among the AT group. Although the AT group had higher CIMT values on both sides (*p* < 0.05) than the age-matched Non-AT group, there was no significant difference in the other surrogate atherosclerosis marker, baPWV, between the two groups. The baPWV is associated with age, blood pressure and eGFR (29). And the AT group and the non-AT group showed no significant differences in all of these (the eGFR was not shown), which may account for the non-significance of baPWV between the two groups.

### Immune and Atherosclerosis Pathways Are Significantly Upregulated in the AT Group

**Figure 2:**
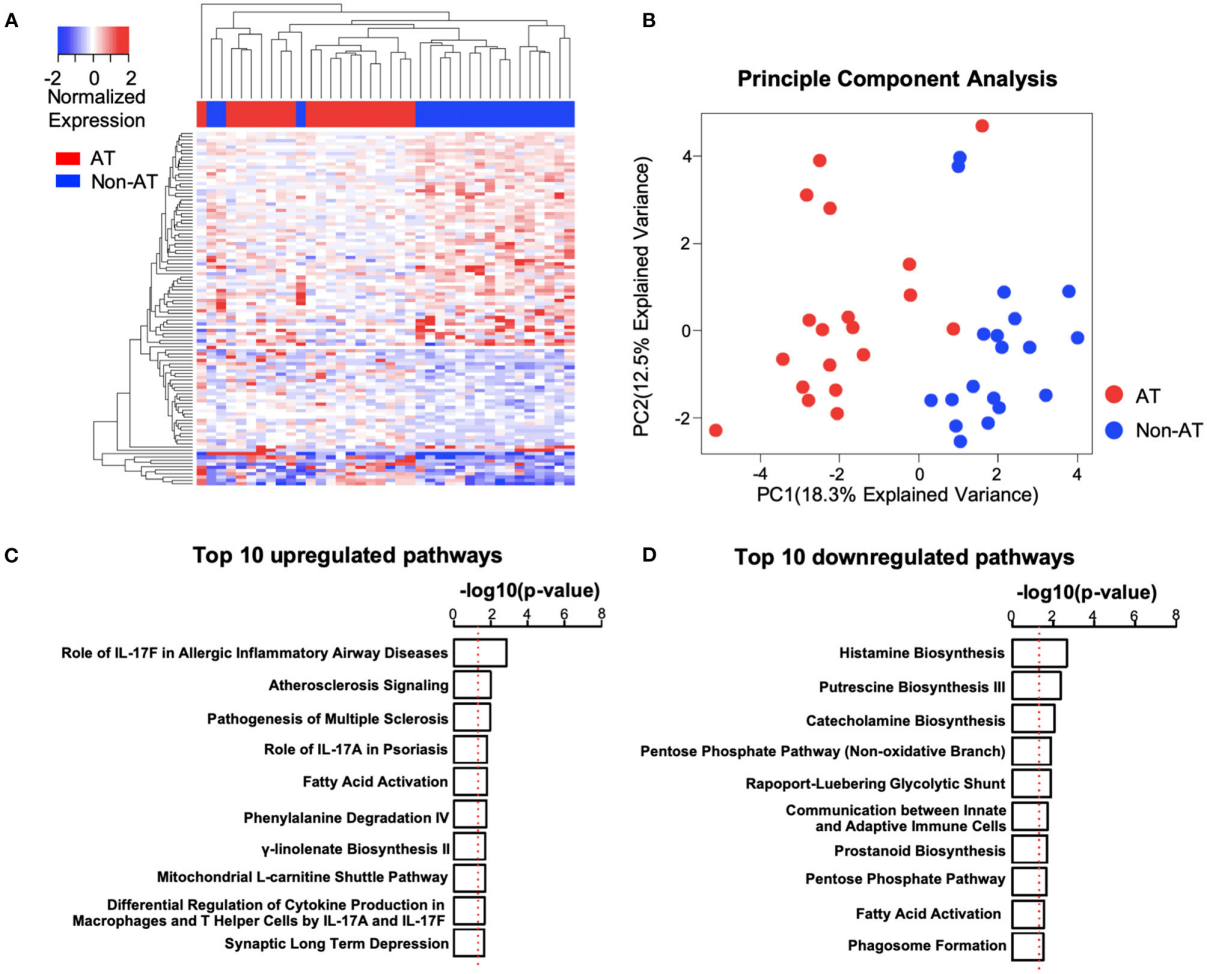
Systemic lupus erythematosus (SLE) atherosclerosis (AT) group has activated atherosclerosis signaling and interleukin (IL)17-related immune pathways. **(A,B)** Heatmap **(A)** and principal component analysis (PCA) plot **(B)** of differentially expressed (DE) genes. The clustering of genes and patients in heatmap was based on complete-linkage, Euclidean distance hierarchical clustering. Red, AT. Blue, Non-AT. **(C,D)** Top 10 upregulated **(C)** and downregulated **(D)** pathways in SLE AT group. Ranked by –log_10_(*p*-value). –log_10_(0.05) is marked with a red line. Ingenuity Pathway Analysis (IPA) was performed with upregulated (*n* = 39) and downregulated (*n* = 67) DE genes.

The differential expression analysis between the age-matched AT and Non-AT groups identified a total of 106 DE genes (Supplementary Table 3), of which 39 were upregulated and 67 were downregulated in the AT group. Uncorrected *p*-values were used to identify DE genes. The principal component analysis and heatmap (Figure 2) showed that patients in the AT group had similar gene expression patterns and that the 106 DE genes could clearly distinguish the AT group from the Non-AT group.

To better understand the differences between the AT and Non-AT groups, we performed an IPA and GSEA on the DE genes. The IPA (Figure 2) showed that “Atherosclerosis Signaling” and multiple IL-17-related pathways (“Role of IL-17F in Allergic Inflammatory Airway Diseases,” “Role of IL-17A in Psoriasis,” “Differential Regulation of Cytokine Production in Macrophages and T Helper Cells by IL-17A and IL-17F”) were significantly activated in the AT group, while multiple metabolic synthesis-related pathways (“Histamine Biosynthesis,” “Putrescine Biosynthesis III,” “Catecholamine Biosynthesis,” and “Prostanoid Biosynthesis”) were inhibited. The GSEA with the KEGG pathways (Supplementary Tables 4, 5) indicated that the pathways related to ribosomes (“KEGG Ribosome”), cell redox (“KEGG Oxidative Phosphorylation”), and cell cycle (“KEGG Cell Cycle”) were upregulated in the AT group, while T cell and B cell receptor pathways (“KEGG T Cell Receptor Signaling Pathway,” “KEGG B Cell Receptor Signaling Pathway”) were significantly downregulated.

To verify the activation of the atherosclerosis pathway in the AT group, we utilized the atherosclerosis signatures reported in previous studies to perform a GSEA (26, 27). In accordance with our expectation, “Huang 2011-Up Genes” were significantly upregulated, and the “Huang 2011-Down Genes” were downregulated in the AT group (Supplementary Figure 1). Up-regulated and down-regulated trends were also identified separately for “Nuhrenberg 2013-Up Genes” and “Nuhrenberg 2013-Down Genes.” These findings confirm that the AT group had increased atherosclerosis signaling.

Because multiple immune pathways showed differences between the AT and Non-AT groups, we further performed a GSEA using immune signatures specific to individual immune cell lineages (25). We observed increased natural killer (NK) cell and CD8^+^ T cell signatures in the AT group compared with the Non-AT group (Supplementary Figure 2), while B cell signatures were decreased, consistent with the downregulation of the B cell receptor pathway in our pathway analysis (Supplementary Table 5).

### Establishment and Evaluation of Risk Prediction Models for Atherosclerosis in Systemic Lupus Erythematosus

We selected five candidate variables from the DE genes and reported clinical risk factors (marked with # in Table 1) for LASSO regression, including three clinical risk factors (age, hyperlipidemia, statin use) and expression levels of two genes (KRT10 and TNNT3) (Supplementary Figure 3). Because of the relative small sample size, we performed the LASSO regression in all SLE patients (*n* = 67), according to the (TRIPOD) statement (28). Multivariate logistic regression showed that KRT10 was significantly related to atherosclerosis in SLE patients (*p* < 0.05) (Supplementary Table 6). Statistical significance and clinical significance were both taken into consideration when we sequentially selected the five candidate variables. Statin use was removed because of its high *p*-value, and the *TNNT3* gene was dropped because it is expressed mainly in skeletal muscle tissue and has little to do with atherosclerosis. After removing statin use and TNNT3, the variables with the highest *p*-values, the remaining three variables (age, hyperlipidemia, KRT10) were all significant in the multivariate logistic regression and were included in the final prediction model (Table 2).

**Table 2:** The estimated coefficients in the prediction model.

**Variable**	**Coefficient**	**95% Confidence interval**	***p*-value**
KRT10	6.10	1.09–11.12	0.017
Hyperlipidemia	2.23	0.35–4.10	0.020
Age	0.12	0.03–0.21	0.012
Intercept	−43.53	−74.60 to −12.45	N/A

*Coefficients of the three variables were calculated with multivariate logistic regression. The final logistic prediction model: The predicted probability of AT = 1/{1+exp[-(−43.53+6.10 × KRT10 expression level+2.23 × hyperlipidemia+0.12 × age)]}. The KRT10 expression level was the DESeq2 vst transformed raw counts. Hyperlipidemia (yes = 1, no = 0), age (years)*.

**Figure 3:**
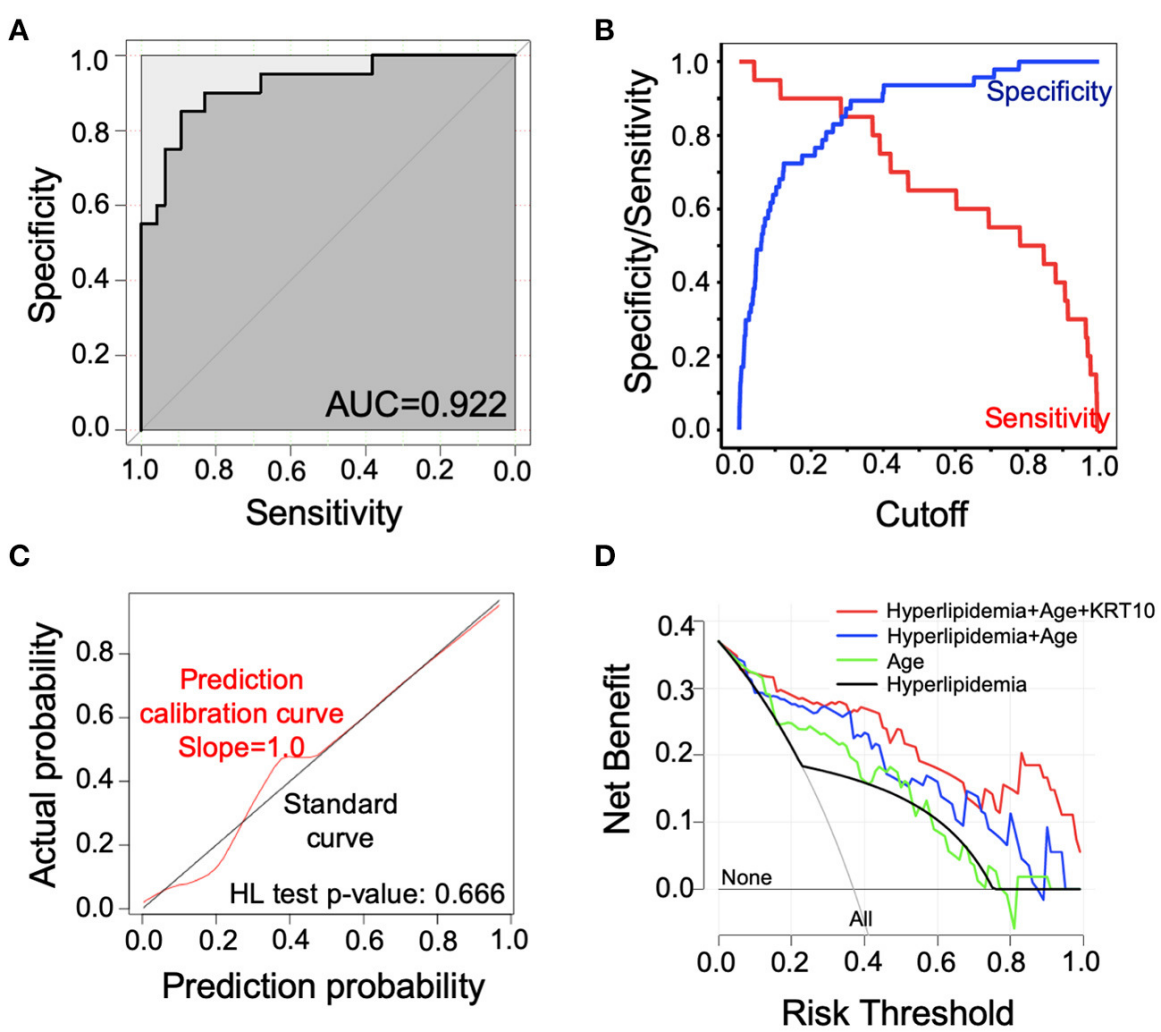
Evaluation of the performance of the risk prediction model. **(A)** Receiver operating characteristic (ROC) curve of the prediction model. **(B)** Sensitivity and specificity of the prediction model with different cutoffs. **(C)** Calibration curve for the logistic regression model. The calibration curve was plotted with 1,000 times bootstrap resampling. *p*-value was calculated with Hosmer–Lemeshow (HL) goodness-of-fit test. **(D)** Decision curve analysis (DCA) comparing net benefit of different models. The net benefit of our prediction model (red line) was compared to models built by only hyperlipidemia (black line), only age (green line), and hyperlipidemia plus age (blue line). The lines labeled with “None” or “All” showed the net benefit of not treating any patients or treating all patients, respectively.

We carried out ROC curve, calibration curve, and decision curve analyses to evaluate the performance of the risk prediction model. The AUC was 0.922 (95% confidence interval, 0.866–0.978, by 500 times bootstrap resampling; Figure 3). As shown in Supplementary Table 7, the prediction model had different sensitivity and specificity at different cutoffs, and the best cutoff in the model was 0.3 points, with a sensitivity of 85.0% and specificity of 87.2% (Supplementary Table 7). The calibration plot (Figure 3) showed relatively high agreement between the prediction by the model and the actual observation in terms of atherosclerosis rate, and the Hosmer–Lemeshow goodness-of-fit test had a vale of *p* = 0.666, suggesting an appreciable discrimination and calibration performance of the prediction model. We compared the net benefits of our prediction model and the models built with age, hyperlipidemia, or age plus hyperlipidemia and found the combination of age, hyperlipidemia, and KRT10 in our model had the highest net benefit (Figure 3). These data suggest that the prediction model had adequate performance and could effectively improve the clinical benefits for the SLE patients regarding atherosclerosis diagnosis and treatment.

## Discussion

In this study, we performed high-throughput RNA-seq and differential expression analysis in SLE patients with and without atherosclerosis. We found that atherosclerosis signaling, CD8^+^ T cell and NK cell signaling, and multiple IL-17-related pathways were significantly activated in SLE patients with atherosclerosis. By combining the DE genes with clinical factors, we constructed a diagnostic prediction model with good performance in predicting the presence of atherosclerosis in patients with SLE. To our knowledge, this is the first study to develop a prediction model for atherosclerosis in SLE patients using high-throughput RNA-seq data.

As well as traditional cardiovascular risk factors, atherosclerosis in SLE is also related to SLE-related risk factors like gene and protein expressions. Recently, several high-throughput RNA-seq analyses have been performed to further investigate the atherosclerosis process in SLE at the gene expression level (12, 13). Korman et al. (13) examined gene expressions during monocyte-to-macrophage differentiation in 20 SLE patients and 16 healthy controls. They identified 3,044 monocyte-to-macrophage differentiation-related genes, among which only 163 genes showed significant differential expression between SLE patients with and without atherosclerosis. These 163 DE genes failed to differentiate SLE patients by atherosclerosis status. Carlucci et al. (12) investigated gene expressions in SLE patients with and without high vascular inflammation or non-calcified plaque burden (NCB), indicators of early vascular disease and plaque vulnerability and rupture risks, respectively. They found that SLE patients with high vascular inflammation (*n* = 9) had increased IFN signaling but downregulated B cell development and T cell and B cell signaling. For SLE patients with high NCB (*n* = 9), NK cell signaling and cross talks between dendritic cells and NK cells were activated, and the NCB was significantly associated with a group of proinflammatory neutrophils/low-density granulocytes. Compared with these two studies, our analyses had a relatively higher number of patients and may add value toward a better understanding of atherosclerosis in SLE.

Our study identified multiple pathways that were differentially regulated in the SLE AT group, among which atherosclerosis signatures were activated. The atherosclerosis signatures in our IPA and from Huang et al. (26) and Nuhrenberg et al. (27) were developed from the general population. This suggests that, despite the identification of many atherosclerosis risk factors and genes specific to SLE (3, 10, 11), SLE patients at least partly share similar atherosclerosis signaling characteristics to the normal population. However, because we did not set a non-SLE control group in the present study, we were unable to determine which of the differentially regulated pathways were specific to SLE. As shown in Table 1, the patients in the AT group in our study were more hyperlipidemic and had a higher statin dosage.

Immune cells play an important role in the development of atherosclerosis (30, 31). In this study, we found that the AT group had increased CD8^+^ T cell and NK cell signatures but decreased B cell signatures. CD8^+^ T cells were reported to promote atherosclerosis progression by inducing inflammation and the formation of necrotic cores in atherosclerotic plaques (32, 33), while the function of NK cells in atherosclerotic plaque formation remained unclear. While previous studies showed that NK cells were pro-atherogenic (33, 34), recent studies demonstrated that few NK cells were identified in atherosclerotic lesions and suggested that NK cells may be irrelevant for atherosclerosis (35, 36). Our findings that NK cell signatures were enriched in the AT group may indicate a potential role of NK cells for the atherosclerosis in SLE. The role of B cells in atherosclerosis has been widely investigated. Different subsets of B cells have different functions in atherosclerosis, with B1 cells being anti-atherosclerotic and B2 cells being pro-atherosclerotic (30). Caligiuri et al. (37) and Major et al. (38) observed an increased incidence of atherosclerosis in B cell-deficient mice, suggesting that B cells exhibit an overall inhibitory effect against atherosclerosis, consistent with the decreased B cell signatures identified in the AT group. In contrast to the reported increased activity of CD4^+^ T cells, dendritic cells, and macrophages reported in atherosclerosis (31), no significant differences in the signatures of these cells were observed between the AT and Non-AT groups. Although these findings may suggest that these immune cells have different roles in the atherosclerosis between SLE patients and the general population, they are more likely to reflect the cells we sequenced, which were from peripheral blood rather than from atherosclerotic lesions. Besides, the pathway analysis revealed that IL-17-related pathways were upregulated in the AT group. IL-17 is important for T helper 17 cells to promote atherosclerosis, and the size of atherosclerotic lesions in IL-17-deficient mice was decreased by 46% (39–41). The activation of IL-17-related pathways in our study suggests that CD4^+^ T cell subsets (such as T helper 17 cells) may be increased in the SLE AT group. Of note, all the immune cell component analyses in this study were based on gene expression data, and the changes in the composition of immune cells need to be verified by fluorescence-activated cell sorting (FACS) or single-cell sequencing.

Early detection and intervention of atherosclerosis are important for the prevention of future CVD. Vascular ultrasound is typically the first-line approach in the detection of atherosclerosis. However, the accuracy of ultrasound is significantly dependent on the experience of the examiner. Our prediction model here can provide an alternative method that is less dependent on the skills of the examiner and may thus be of benefit to SLE patients in developing countries with a lack of good healthcare. Furthermore, the model can be applied in large-scale screening and rountine clinical use and may assist clinicians to better detect subclinical atherosclerosis. To establish our prediction model, we combined DE genes identified by high-throughput RNA-seq and important clinical risk factors. Three variables were used in the final prediction model, among which age and hyperlipidemia are classic risk factors for atherosclerosis. KRT10 is a member of the cytokeratin family. A previous study showed that increased expressions of cytokeratin 8, 18, and 19 were related to increased arterial intimal thickening features in atherosclerosis (42), while a link between KRT10 and atherosclerosis has not been reported. The present data suggest that KRT10 may play a role in the atherosclerosis in SLE. Further investigations are required to clarify whether KRT10 is a driver or simply a marker for atherosclerosis.

Here, we utilized LASSO regression to build the prediction model, which could properly eliminate the multicollinearity among independent variables and reduce model complexity by parameter selection. Compared with the prediction models containing only age (AUC = 0.826), only hyperlipidemia (AUC = 0.722), or combination of age and hyperlipidemia (AUC = 0.880), addition of KRT10 to the model significantly increased the prediction performance (AUC = 0.922) and benefits for clinical decision-making (Figure 3). The PREDICTS model for atherosclerosis in SLE was developed by McMahon et al. (17) based on a western population. Their model utilized proinflammatory HDL (piHDL), leptin, TWEAK, homocysteine, history of diabetes, and age as predictors and had a sensitivity of 89% and specificity of 79% (AUC not available). With fewer variables (3 vs. 6), our model had a relatively lower sensitivity of 85% (0.3 as cutoff; Figure 3, Supplementary Table 7) than the PREDICTS model but a higher specificity of 87%. Furthermore, because Asian SLE populations have different prevalence of CVD and genetic backgrounds from western populations (43, 44), whether the PREDICTS model is applicable to the Chinese population remains to be confirmed. Thus, with better predictive capacity and Asian population specificity, this prediction model can serve as a useful tool in identifying atherosclerosis risk in Asian SLE patients early.

There are some limitations to our study. Firstly, our study included only Chinese individuals, and the number of samples was relatively small, which may probably limit the application of the prediction model. And with this small sample size, certain atherosclerosis-related genes may not be identified as DE genes. There was imbalance of classes in the dataset (20:47) because of the relative small sample size. While the sample size met the modeling requirements of the prediction model based on the internal verification of our study, it still limited the application of the prediction model in a larger population. And how the prediction model will perform in a larger sample size remains to be studied. Moreover, the patients in the AT group in our study were more hyperlipidemic and had a higher statin dosage, which could have effects on cellular lipid metabolism and other transcriptomic pathways. Secondly, as shown in Supplementary Table 1, our study population had long disease durations of SLE, and none of them was in active phase when their clinical information and blood were collected. Moreover, organ damage was observed in both AT and non-AT groups. These characteristics of the enrolled patients may reduce the representativeness. Thirdly, owing to the unavailability of clinical information or gene expression data in published studies, we could not perform external validation of our prediction model. Thus, the accuracy of our model generated from a small population will need to be further validated when it is applied to a greater population. Finally, the study had a cross-sectional design, and thus the causal relationships between DE genes like KRT10 and atherosclerosis could not be determined. Further studies are required to determine whether this model can predict the future risk of atherosclerosis.

## Conclusion

In conclusion, we have built an atherosclerotic risk prediction model utilizing one gene and two clinical factors that can effectively predict the presence of atherosclerosis in Chinese SLE patients. This model may assist clinicians to identify SLE patients with a high risk of atherosclerosis.

## Data Availability Statement

The data has been deposited at Genome Sequence Archive (https://bigd.big.ac.cn/gsa-human/browse/HRA000525) with accession number: HRA000525.

## Ethics Statement

The studies involving human participants were reviewed and approved by The Institutional Review Board of Peking Union Medical College Hospital. The patients/participants provided their written informed consent to participate in this study.

## Author Contributions

SZ, ML, HP, TD, and HX contributed to the study conception and design. Blood samples and clinical data were collected by TD, HX, and XY. JZ measured the carotid intima media thickness. TD and HP performed the statistical analysis. The first draft of the manuscript was written by TD and HX. ML, HP, and SZ provided critical revision for the manuscript. All authors read and approved the final manuscript.

## Conflict of Interest

The authors declare that the research was conducted in the absence of any commercial or financial relationships that could be construed as a potential conflict of interest.

## Abbreviations

SLE: systemic lupus erythematosus
AT: atherosclerosis
DE: differentially expressed
CVD: cardiovascular disease
CIMT: carotid intima media thickness
IPA: Ingenuity Pathway Analysis
GSEA: gene set enrichment analysis
LASSO: least absolute shrinkage and selection operator
ROC: receiver operating characteristics
AUC: area under the curve
SLEDAI: SLE Disease Activity Index 2000
baPWV: brachial-ankle pulse wave velocity
SLICC/ADI: Systemic Lupus International Collaborating Clinics/ACR Damage Index
PREDICTS: Predictors of Risk for Elevated Flares, Damage Progression, and Increased Cardiovascular Disease in Patients with SLE.
