# Supplementary material for: Establishing a Risk Prediction Model for Atherosclerosis in Systemic Lupus Erythematosus
Source: Front Immunol. 2021 Apr 16;12:622216. doi: 10.3389/fimmu.2021.622216 (PMC8085548; doi:10.3389/fimmu.2021.622216)
Supplement: Supplementary file 1 [file Data_Sheet_1.docx]

**
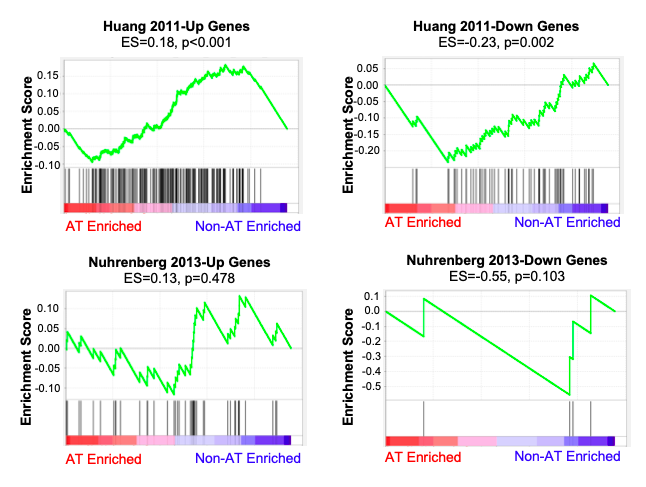
**

**Figure S1. Gene set enrichment analysis of atherosclerosis signatures in SLE AT group.**

Atherosclerosis signatures were from Huang et al. (18) and Nuhrenberg et al. (19). “Up genes” and “down genes” represent genes up- or down-regulated in atherosclerosis. Genes used in GSEA were ranked by log2(fold change).


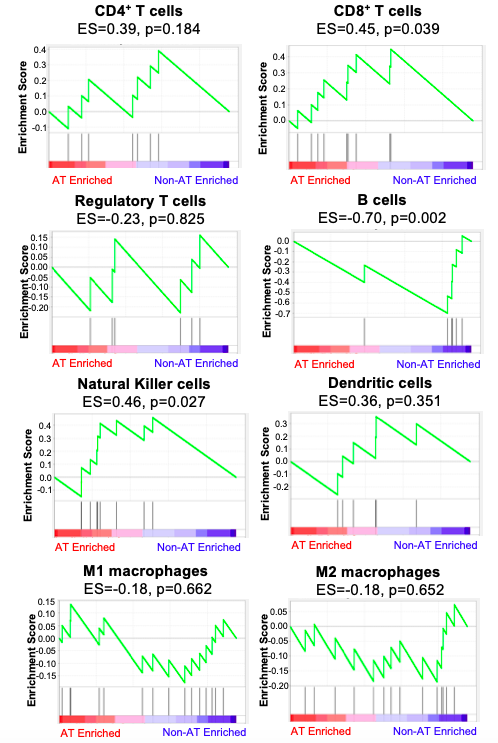


**Figure S2. Gene set enrichment analysis of immune cell signatures in SLE AT group.**

Immune cell signatures were developed by Davoli et al. (20) with immune genes specific to different immune cell lineages. Genes used in GSEA were ranked by log_2_(fold change).


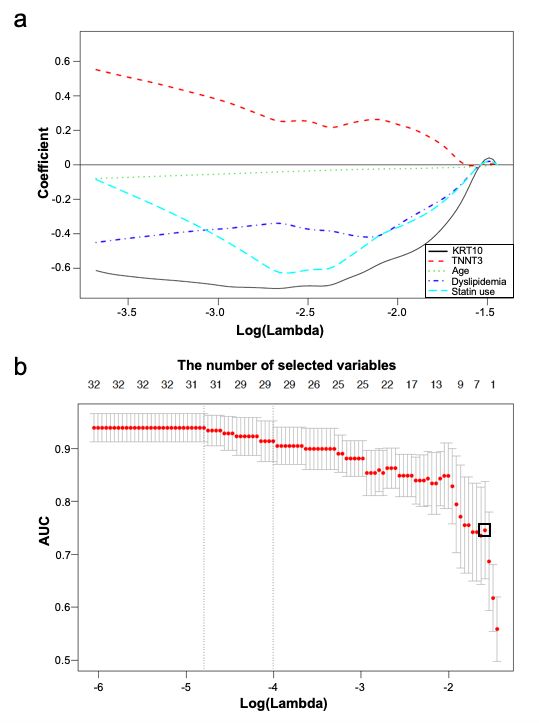


**Figure S3. Selection of features by Lasso.**

(A) The coefficients of the variables at different lambda. (B) The area under ROC curve (AUC) at different lambda. Each lambda had a corresponding set of selected variables and AUC. The lambda with 5 candidate variables was labeled by black square.

**Table S1. Demographics and clinical features of all 67 SLE patients**

| Factor | SLE  (N=67) |
| --- | --- |
| Age (years) | 42.7(36.6-51.2) |
| Male, n (%) | 4(6.0) |
| BMI (kg/m^2^) | 23.6(21.5-26.0) |
| Disease duration of SLE, (years) | 10.0(6.6-15.2) |
| Ever smoker, n (%) | 4(6.0) |
| Menopausal status, n (%) | 34(50.7) |
| Hypertension, n (%) | 14(20.9) |
| Hyperlipidemia, n (%) | 16(23.9) |
| Diabetes mellitus, n (%) | 5(7.5) |
| Family history of early onset CVD, n (%) | 17(25.4) |
| Coronary heart disease, n (%) | 4(6.0) |
| Stroke, n (%) | 1(1.5) |
| Atherosclerosis, n (%) | 20(29.9) |
| SLEDAI | 2(0-2) |
| SLICC/ADI | 0(0-1) |
| Positive Anti-dsDNA antibodies, n (%) | 31(46.3) |
| Aspirin, n (%) | 10(14.9) |
| Statins, n (%) | 6(9.0) |
| ARB/ACEI, n (%) | 10(14.9) |
| Corticosteroids, n (%) | 39(58.2) |
| Current use of prednisone (mg) | 2.5(0-5.0) |
| 12-month cumulative prednisone (g) | 0.91(0-1.83) |
| Hydroxychloroquine, n (%) | 55(82.1) |
| Cyclophosphamide, n (%) | 3(4.5) |
| Azathioprine, n (%) | 5(7.5) |
| Cyclosporine, n (%) | 2(3.0) |
| Tripterygium wilfordii, n (%) | 1(1.5) |
| Mycophenolate mofetil, n (%) | 6(9.0) |

Note: SLEDAI: systemic lupus erythematosus disease activity index 2000; SLICC/ADI: Systemic Lupus International Collaborating Clinics/ACR Damage Index; ARB/ACEI: angiotensin~converting enzyme inhibitor/angiotensin receptor blocker. Data were presented as median and quartiles (Q1-Q3) for continuous variables and as percentages (n/N) for categorical variables.

**Table S2. Demographics and clinical features of AT and Non-AT groups**

| Factor | Atherosclerosis  (N=20) | Non-atherosclerosis  (N=47) | P value |
| --- | --- | --- | --- |
| Age (years) | 52.4(49.3-58.6) | 39.7(34.55-47.95) | <0.001^*^ |
| Male, n (%) | 1(5.0) | 3(6.4) | 1.000 |
| BMI (kg/m^2^) | 24.5(23.6-26.7) | 22.9(21.3-25.4) | 0.054 |
| Disease duration of SLE, (years) | 10.1(6.6-17.6) | 10.0(6.6-14.3) | 0.848 |
| Ever smoker, n (%) | 3(15.0) | 3(6.4) | 0.507 |
| Menopausal status, n (%) | 15(75.0) | 19(40.4) | 0.013 |
| Hypertension, n (%) | 9(45.0) | 5(10.6) | 0.005^*^ |
| Hyperlipidemia, n (%) | 11(55.0) | 5(10.6) | <0.001^*^ |
| Diabetes mellitus, n (%) | 2(10.5) | 2(10.5) | 1.000 |
| Family history of early onset CVD, n (%) | 3(15.0) | 2(4.3) | 0.306 |
| Coronary heart disease, n (%) | 9(45.0) | 8(17.0) | 0.036^*^ |
| Stroke, n (%) | 4(20.0) | 0(0) | 0.009^*^ |
| TC (mmol/L) | 1(5.0) | 0(0) | 0.657 |
| TG (mmol/L) | 4.29(4.04-4.70) | 4.09(3.59-4.50) | 0.170 |
| HDL-C (mmol/L) | 1.19(1.06-1.51) | 1.21(1.10-1.47) | 0.831 |
| LDL-C (mmol/L) | 2.50(1.91-3.03) | 2.31(1.91-2.74) | 0.253 |
| WBC (×10^9^) | 5.61(3.93-7.79) | 4.76(3.87-5.85) | 0.415 |
| NEUT (×10^9^) | 3.28(1.87-4.77) | 3.23(2.34-3.95) | 0.795 |
| LYM (×10^9^) | 1.77(1.17-2.15) | 1.28(1.00-1.83) | 0.087 |
| PLT (×10^9^) | 217(150-267) | 219(176-253) | 0.713 |
| HbA1c (%) | 5.5(5.2-5.9) | 5.3(5.1-5.5) | 0.029^*^ |
| FBG (mmol/L) | 4.8(4.6-5.0) | 4.5(4.4-4.7) | 0.010^*^ |
| Cr(μmol/L) | 65(61-73) | 59(52-67) | 0.005^*^ |
| UA(μmol/L) | 286(245-338) | 296(264-338) | 0.429 |
| C3(g/L) | 0.974  (0.931-1.327) | 0.949  (0.831-1.047) | 0.113 |
| C4(g/L) | 0.181  (0.144-0.201) | 0.138  (0.098-0.175) | 0.033^*^ |
| ESR (mm/h) | 11(9-24) | 18(10-25) | 0.491 |
| hs-CRP (mg/L) | 1.16(0.71-4.81) | 1.47(0.64-2.58) | 0.823 |
| CK (U/L) | 72(58-97) | 69(56-96) | 0.893 |
| CK-MB (μg/L) | 0.5(0.5-0.6) | 0.5(0.5-0.6) | 0.463 |
| cTnI (μg/L) | <0.017 | <0.017 | 0527 |
| NT-proBNP (pg/ml) | 55(36-91) | 48(24-84) | 0.640 |
| Anti-dsDNA antibodies, n (%) | 7(35.0) | 24(51.1) | 0.271 |
| Left CIMT (mm) | 1.52(0.73-1.80) | 0.65(0.56-0.78) | <0.001^*^ |
| Right CIMT (mm) | 1.20(0.79-1.73) | 0.62(0.56-0.74) | <0.001^*^ |
| Left baPWV (cm/s) | 1528  (1294-1701) | 1250  (1151-1429) | 0.006^*^ |
| Right baPWV (cm/s) | 1520  (1317-1684) | 1276  (1194-1472) | 0.006^*^ |
| SLEDAI | 2(0-2) | 2(0-2) | 0.588 |
| SLICC/ADI | 0(0-1) | 0(0-0) | 0.010^*^ |
| Aspirin, n (%) | 5(25.0) | 5(10.6) | 0.256 |
| Statins, n (%) | 6(30.0) | 0(0) | <0.001^*^ |
| ARB/ACEI, n (%) | 6(30.0) | 4(8.51) | 0.060 |
| Corticosteroids, n (%) | 11(55.0) | 28(59.6) | 0.939 |
| Current use of prednisone (mg) | 1.0(0-5.0) | 2.5(0-5.0) | 0.348 |
| 12-month cumulative prednisone (g) | 0.68(0-1.83) | 0.91(0-2.74) | 0.298 |
| Hydroxychloroquine, n (%) | 17(85.0) | 38(80.9) | 0.954 |
| Cyclophosphamide, n (%) | 2(10.0) | 1(2.1) | 0.435 |
| Azathioprine, n (%) | 2(10.0) | 3(6.4) | 0.994 |
| Cyclosporine, n (%) | 0(0) | 2(4.3) | 0.879 |
| Tripterygium wilfordii, n (%) | 0(0) | 1(2.1) | 1.000 |
| Mycophenolate mofetil, n (%) | 0(0) | 6(12.8) | 0.227 |

Notes: AT, atherosclerosis; Non-AT: patients without atherosclerosis; TC: total cholesterol; TG: triglyceride; LDL-C: low-density lipoprotein cholesterol; HDL-C: high-density lipoprotein cholesterol; WBC: No. of white blood cell; NEUT: No. of neutrophil; LYM: No. of lymphocyte; PLT: No. of platelet; HbA1c: glycated hemoglobin A1c; FBG: fasting blood glucose; Cr: creatinine; UA: uric acid; C3: complement 3; C4: complement 4; ESR: erythrocyte sedimentation rate; hs-CRP: high sensitivity C-Reactive Protein; CK: creatine kinase; CK-MB: creatine kinase isoenzyme; cTnI: cardiac troponin I; NT-proBNP: N-terminal pro-B-type natriuretic peptide; dsDNA: double strand DNA; CIMT: carotid intima-media thickness; baPWV: the brachial-ankle pulse wave velocity; SLEDAI: systemic lupus erythematosus disease activity index 2000; SLICC/ADI: Systemic Lupus International Collaborating Clinics/ACR Damage Index; ARB/ACEI: angiotensin~converting enzyme inhibitor/angiotensin receptor blocker. Data were presented as median and quartiles (Q1-Q3) for continuous variables and as percentages (n/N) for categorical variables. Comparisons between the two groups were conducted with Wilcoxon rank-sum test and chi-square test for continuous variables and categorical variables, respectively. *p value<0.05.

**Table S3. Differentially expressed genes**

| Ensembl ID | Gene symbol | log2FoldChange  AT vs Non-AT | p-value |
| --- | --- | --- | --- |
| ENSG00000121335 | PRB2 | 4.45 | 0.0019 |
| ENSG00000275290 | LILRA2 | 3.91 | 0.0001 |
| ENSG00000047617 | ANO2 | 2.63 | 0.0003 |
| ENSG00000239754 | CFB | 2.26 | 0.0017 |
| ENSG00000230162 | CT45A11P | 2.18 | 0.0009 |
| ENSG00000220721 | OR1F12 | 2.08 | 0.0030 |
| ENSG00000275302 | CCL4 | 2.02 | <0.0001 |
| ENSG00000183929 | DUSP5P1 | 2.01 | 0.0064 |
| ENSG00000164684 | ZNF704 | 2.00 | 0.0049 |
| ENSG00000227257 | AL158827.1 | 2.00 | 0.0064 |
| ENSG00000143341 | HMCN1 | 1.76 | 0.0002 |
| ENSG00000160460 | SPTBN4 | 1.74 | 0.0036 |
| ENSG00000104918 | RETN | 1.67 | 0.0010 |
| ENSG00000256618 | MTRNR2L1 | 1.65 | 0.0006 |
| ENSG00000017427 | IGF1 | 1.47 | 0.0056 |
| ENSG00000163221 | S100A12 | 1.34 | <0.0001 |
| ENSG00000101425 | BPI | 1.24 | 0.0098 |
| ENSG00000140284 | SLC27A2 | 1.24 | 0.0004 |
| ENSG00000255492 | AC104383.1 | 1.21 | 0.0063 |
| ENSG00000187980 | PLA2G2C | 1.19 | 0.0023 |
| ENSG00000152766 | ANKRD22 | 1.19 | 0.0077 |
| ENSG00000165338 | HECTD2 | 1.08 | 0.0049 |
| ENSG00000254681 | PKD1P5 | 1.08 | 0.0049 |
| ENSG00000150760 | DOCK1 | 1.06 | 0.0054 |
| ENSG00000101132 | PFDN4 | 1.04 | 0.0071 |
| ENSG00000232871 | SEC1P | 1.01 | 0.0086 |
| ENSG00000050730 | TNIP3 | 0.99 | 0.0019 |
| ENSG00000169607 | CKAP2L | 0.99 | 0.0059 |
| ENSG00000104611 | SH2D4A | 0.92 | 0.0016 |
| ENSG00000109475 | RPL34 | 0.92 | 0.0071 |
| ENSG00000143546 | S100A8 | 0.89 | 0.0061 |
| ENSG00000240024 | LINC00888 | 0.86 | 0.0060 |
| ENSG00000196074 | SYCP2 | 0.83 | 0.0024 |
| ENSG00000282204 | TRBV9 | 0.77 | 0.0073 |
| ENSG00000116661 | FBXO2 | 0.76 | 0.0079 |
| ENSG00000177301 | KCNA2 | 0.75 | 0.0092 |
| ENSG00000214954 | LRRC69 | 0.67 | 0.0067 |
| ENSG00000270518 | AC008626.1 | 0.62 | 0.0090 |
| ENSG00000186395 | KRT10 | 0.61 | 0.0023 |
| ENSG00000142920 | AZIN2 | -0.61 | 0.0037 |
| ENSG00000168878 | SFTPB | -0.61 | 0.0030 |
| ENSG00000198182 | ZNF607 | -0.61 | 0.0059 |
| ENSG00000234776 | C11orf94 | -0.61 | 0.0065 |
| ENSG00000171219 | CDC42BPG | -0.61 | 0.0010 |
| ENSG00000183578 | TNFAIP8L3 | -0.61 | 0.0096 |
| ENSG00000189401 | OTUD6A | -0.64 | 0.0040 |
| ENSG00000182389 | CACNB4 | -0.71 | 0.0078 |
| ENSG00000181616 | OR52H1 | -0.72 | 0.0078 |
| ENSG00000241322 | CDRT1 | -0.75 | 0.0075 |
| ENSG00000182240 | BACE2 | -0.76 | 0.0055 |
| ENSG00000282379 | DTX2 | -0.77 | 0.0023 |
| ENSG00000269404 | SPIB | -0.78 | 0.0078 |
| ENSG00000164440 | TXLNB | -0.78 | 0.0038 |
| ENSG00000224722 | AC020688.1 | -0.86 | 0.0026 |
| ENSG00000233579 | KRT8P15 | -0.91 | 0.0070 |
| ENSG00000007350 | TKTL1 | -0.93 | 0.0093 |
| ENSG00000115718 | PROC | -0.93 | 0.0029 |
| ENSG00000131016 | AKAP12 | -0.93 | 0.0004 |
| ENSG00000288250 | TNNT3 | -0.97 | 0.0039 |
| ENSG00000070018 | LRP6 | -1.00 | 0.0076 |
| ENSG00000153904 | DDAH1 | -1.00 | 0.0008 |
| ENSG00000117322 | CR2 | -1.01 | 0.0030 |
| ENSG00000196302 | AC146944.1 | -1.01 | 0.0010 |
| ENSG00000174370 | C11orf45 | -1.01 | 0.0077 |
| ENSG00000130377 | ACSBG2 | -1.01 | 0.0062 |
| ENSG00000140287 | HDC | -1.04 | 0.0080 |
| ENSG00000250677 | AC114781.3 | -1.06 | 0.0032 |
| ENSG00000167244 | IGF2 | -1.10 | 0.0088 |
| ENSG00000143369 | ECM1 | -1.21 | 0.0002 |
| ENSG00000276192 | IGHE | -1.26 | 0.0045 |
| ENSG00000274513 | LILRB2 | -1.26 | 0.0011 |
| ENSG00000128250 | RFPL1 | -1.27 | 0.0094 |
| ENSG00000133101 | CCNA1 | -1.31 | 0.0078 |
| ENSG00000137204 | SLC22A7 | -1.36 | 0.0026 |
| ENSG00000271010 | AC011475.1 | -1.37 | 0.0003 |
| ENSG00000266728 | AC015688.4 | -1.38 | 0.0062 |
| ENSG00000284746 | AC068587.8 | -1.42 | 0.0059 |
| ENSG00000163106 | HPGDS | -1.42 | 0.0017 |
| ENSG00000237988 | OR2I1P | -1.44 | 0.0064 |
| ENSG00000169744 | LDB2 | -1.45 | 0.0094 |
| ENSG00000116254 | CHD5 | -1.47 | 0.0023 |
| ENSG00000163092 | XIRP2 | -1.47 | 0.0046 |
| ENSG00000161939 | RNASEK-C17orf49 | -1.48 | 0.0020 |
| ENSG00000156453 | PCDH1 | -1.54 | 0.0001 |
| ENSG00000116117 | PARD3B | -1.56 | 0.0013 |
| ENSG00000204851 | PNMA8B | -1.65 | 0.0006 |
| ENSG00000282092 | MED16 | -1.69 | 0.0016 |
| ENSG00000099957 | P2RX6 | -1.70 | 0.0027 |
| ENSG00000164708 | PGAM2 | -1.73 | 0.0050 |
| ENSG00000167355 | OR51B5 | -1.77 | 0.0020 |
| ENSG00000204196 | RPL12P16 | -1.79 | 0.0040 |
| ENSG00000141744 | PNMT | -1.82 | 0.0031 |
| ENSG00000039560 | RAI14 | -1.87 | 0.0033 |
| ENSG00000128242 | GAL3ST1 | -1.94 | 0.0038 |
| ENSG00000179023 | KLHDC7A | -1.95 | 0.0055 |
| ENSG00000184100 | BRD7P2 | -1.95 | 0.0041 |
| ENSG00000211893 | IGHG2 | -1.98 | <0.0001 |
| ENSG00000094963 | FMO2 | -2.19 | 0.0049 |
| ENSG00000276468 | KIR2DP1 | -2.22 | 0.0062 |
| ENSG00000166535 | A2ML1 | -2.22 | 0.0026 |
| ENSG00000235961 | PNMA6A | -2.27 | 0.0013 |
| ENSG00000183090 | FREM3 | -2.30 | 0.0022 |
| ENSG00000183850 | ZNF730 | -2.37 | 0.0091 |
| ENSG00000278042 | IGHV3-72 | -5.57 | 0.0009 |
| ENSG00000204619 | PPP1R11 | -23.37 | <0.0001 |
| ENSG00000236560 | PPP1R11 | -23.89 | <0.0001 |

**Table S4. The top 10 upregulated pathways in the AT group based on GSEA analysis**

| Pathway | Enrichment Score | P value | FDR |
| --- | --- | --- | --- |
| KEGG Ribosome | 0.54 | <0.001 | <0.001 |
| KEGG Systemic Lupus Erythematosus | 0.33 | <0.001 | <0.001 |
| KEGG Alzheimer’s Disease | 0.24 | <0.001 | <0.001 |
| KEGG Parkinson’s Disease | 0.26 | <0.001 | <0.001 |
| KEGG Oxidative Phosphorylation | 0.25 | <0.001 | <0.001 |
| KEGG Ubiquitin Mediated Proteolysis | 0.20 | <0.001 | <0.001 |
| KEGG Cell Cycle | 0.21 | <0.001 | <0.001 |
| KEGG Spliceosome | 0.22 | <0.001 | <0.001 |
| KEGG Huntington’s Disease | 0.17 | <0.001 | 0.002 |
| KEGG Proteasome | 0.31 | <0.001 | 0.002 |

Notes: 186 KEGG pathways were used to performed GSEA analysis. FDR, false discovery rate.

**Table S5. The top 10 downregulated pathways in the AT group based on GSEA analysis**

| Pathway | Enrichment Score | P value | FDR |
| --- | --- | --- | --- |
| KEGG Endocytosis | -0.16 | <0.001 | 0.007 |
| KEGG B Cell Receptor Signaling Pathway | -0.24 | <0.001 | 0.010 |
| KEGG Fc Gamma R Mediated Phagocytosis | -0.20 | <0.001 | 0.020 |
| KEGG MAPK Signaling Pathway | -0.12 | <0.001 | 0.020 |
| KEGG Lysosome | -0.17 | <0.001 | 0.022 |
| KEGG Apoptosis | -0.20 | 0.002 | 0.022 |
| KEGG Notch Signaling Pathway | -0.25 | 0.002 | 0.033 |
| KEGG T Cell Receptor Signaling Pathway | -0.17 | 0.006 | 0.034 |
| KEGG Acute Myeloid Leukemia | -0.22 | 0.010 | 0.046 |
| KEGG Arachidonic Acid Metabolism | -0.22 | 0.012 | 0.072 |

Notes: 186 KEGG pathways were used to performed GSEA analysis. FDR, false discovery rate.

**Table S6. The coefficients of the KRT10, statin use, hyperlipidemia, age and TNN3 in the multivariate logistic regression analysis**

| Variable | Coefficient | 95% confidence interval | P value |
| --- | --- | --- | --- |
| Statin use | 17.36 | -4582-4616 | 0.994 |
| TNNT3 | -1.67 | -4.54-1.21 | 0.256 |
| Age | 0.08 | -0.02-0.18 | 0.102 |
| Hyperlipidemia | 1.99 | -0.20-4.18 | 0.074 |
| KRT10 | 5.84 | 0.50-11.19 | 0.032 |

**Table S7. The specificity and sensitivity of the prediction model at different cutoffs**

| Cutoff | Sensitivity | Specificity |
| --- | --- | --- |
| 0.1 | 95.0% | 63.8% |
| 0.3 | 85.0% | 87.2% |
| 0.5 | 65.0% | 93.6% |
| 0.7 | 55.0% | 95.7% |
| 0.9 | 40.0% | 100.0% |
